# Association of Voltage-Gated Potassium Channel Polymorphisms with the Risk and Prognosis of Epilepsy in the Saudi Population: A Case–Control Study

**DOI:** 10.3390/medicina61030396

**Published:** 2025-02-25

**Authors:** Mansour A. Alghamdi, Laith N. AL-Eitan, Mansour Y. Otaif, Doaa M. Rababa’h, Maryam K. Alasmar, Abdulaziz M. Al-Garni, Rayyh A. M. Saleh, Nawal F. Abdel Ghaffar

**Affiliations:** 1 Department of Anatomy, College of Medicine, King Khalid University, Abha 62529, Saudi Arabia; 2 Genomics and Personalized Medicine Unit, The Center for Medical and Health Research, King Khalid University, Abha 62529, Saudi Arabia; 3 Department of Biotechnology and Genetic Engineering, Jordan University of Science and Technology, Irbid 22110, Jordan; 4 Department of Pediatric, Neurology Section, Abha Maternity and Children Hospital, Abha 62562, Saudi Arabia; 5 Department of Medicine, College of Medicine, King Khalid University, Abha 62529, Saudi Arabia; amoalqarni@kku.edu.sa; 6 Clinical Pathology, Faculty of Medicine (for Girls), Al-Azhar University, Cairo 11884, Egypt; 7 Laboratory and Blood Bank Department, Aseer Central Hospital, Abha 62523, Saudi Arabia; 8 Neurology Department, Kasr Al Ainy Hospital, Faculty of Medicine, Cairo University, Giza 12613, Egypt; naabdelghaffar@moh.gov.sa; 9 Neurology Department, Aseer Central Hospital, Abha 62523, Saudi Arabia

**Keywords:** epilepsy, genetic polymorphism, *KCNAB1*, *KCNJ10*, potassium channels

## Abstract

*Background and Objectives*: Epilepsy, known as an unprovoked seizure, arises from the human brain. Genetics plays a fundamental role in the development and progression of the disorder. This study aimed to investigate the influence of voltage-gated K^+^ channels on the risk of epilepsy. *Materials and Methods*: Several genetic variants were examined using PCR sequencing. This case–control study was conducted on 296 individuals who were diagnosed with epilepsy, in addition to 293 healthy participants. *Results*: This study revealed that within *KCNAB1*, both rs3755631 and rs4679773 are correlated with epilepsy, and the *p*-values = 0.04 for both allelic associations. In addition, regarding the *KCNJ10* gene, we found that rs2820585, rs946420, rs1186679, rs61822012, and rs1186685 were significantly correlated with epilepsy risk (*p*-values = 0.034, 0.045, 0.021, 0.048, and 0.018), respectively. *Conclusions*: From the current study, we conclude that voltage-gated potassium channels can impact epilepsy risk and can also interfere with the prognosis of epilepsy.

## 1. Introduction

Epilepsy is a common neurological disorder that is characterized by unprovoked seizures generated by an abnormal electrical flow from a specific part of the human brain [1,2]. Approximately 50 million people worldwide are affected by epilepsy, making it one of the most common neurological disorders. Epilepsy can affect both genders of all races and affect people of all ages. However, the prevalence of epilepsy among men is slightly higher than among women [3].

The last documented prevalence study on active epilepsy cases in the Kingdom of Saudi Arabia (KSA) was conducted in 2001, estimating a prevalence of 6.5 per 1000 individuals [4]. Cultural understanding and acceptance of epileptic patients have increased over the years in the Saudi population as more people have become aware of the disease, and general knowledge has increased [5,6]. This led to more acceptance for epileptic patients in different parts of the community and community-based activities [5,6].

The etiology of epilepsy is a significant determinant of the clinical manifestation and prognosis of the disease [7]. Several prominent factors have been identified as part of the etiology of epilepsy. Epilepsy disorder is a complex condition in which both environmental and genetic factors can interfere with the progression and development of the disease [8]. Brain injuries during the early stages of development of people; brain infections; brain trauma; and brain conditions such as stroke, tumor, and blood vessel problems are the commonly recognized causes of epilepsy [9]. Genetic defects can result in an epileptic seizure known as genetic (non-inherited) epilepsy [10]. Furthermore, environmental factors can trigger genetic variation within a person’s genome throughout their life [11]. However, the family history of an individual with epilepsy is a significant risk factor for the development of the disease and can be determined as inherited epilepsy [12].

Potassium (K) channels are crucial in the demonstration of genetic epilepsy. The K channel family is the largest among ion-gated channels and comprises more than 70 human genes that encode different α subunits [13]. These channels are essential for neuronal excitability, and several epileptic phenotypes have been attributed to functional impairment in potassium channels (K^+^) [14]. Voltage-gated K^+^ channels (Kv) regulate action potentials, modulate neurotransmitters, and control electrical properties [15]. They also regulate various pathways of neuronal life and death, such as apoptosis, channel phosphorylation, or cell proliferation [16].

The *KCNA1* gene is located on chromosome 12 and encodes the voltage-gated K^+^ channel subunit Kv1.1 [17]. Several heterozygous point mutations have been identified within this gene that cause loss of function and subsequently alter the properties of the channel and are associated with reduced currents [18]. This alteration results in epileptic phenotypes such as generalized or partial seizures associated with episodic ataxia (EA) [19,20]. On the other hand, a complete loss of *KCNA1* function has been implicated in neonatal epileptic encephalopathy (NEE) [21].

The *KCNA2* gene is located in chromosome 1 and encodes an α subunit of the Kv1.2 channel. This gene has been involved in epileptic encephalopathy (EE) [22], ataxia, and myoclonic epilepsy [23]. Many genetic polymorphisms within *KCNA2* have been extensively investigated, and several were correlated with epilepsy risk and have been reported in patients with BFNIS, GEFS+, and Dravet syndrome [24]. Variant effects on K channel activity fluctuate; some confer loss of function, such as p.Ile263Thr and p.Pro405Leu, while others manifest gain of function, such as p.Arg297Gln and p.Leu298Phe [25]. The voltage-gated potassium channel, Kv8.2, is encoded by *KCNV2*. Kv8.2 can interact functionally with Kv2 subunits, impacting membrane translocation and channel properties [26]. The p. Arg7Lys variant has been reported to influence K channel function by decreasing the delayed rectifier K^+^ current in neurons and is recognized among patients with febrile and febrile partial seizures. Furthermore, p.Met285Arg within *KCNV2* is associated with epileptic encephalopathy and severe refractory epilepsy [20].

*KCNAB2* is another gene encoded for the voltage-gated potassium channel Kvβ2 and has been involved with severe epilepsy. Hemizygous deletion of *KCNAB2* results in loss of function of channel activity [27]. *KCNJ10* and *KCNJ9* have also been correlated with the risk of epilepsy. *KCNJ10* encodes Kir4.1, and p.Arg271Cys within the gene is responsible for susceptibility to seizures, even though it does not confer changes in channel function and structure [28]. p. Arg65Cys is another recessive mutation of the *KCNJ10* gene that causes channel loss of function and is related to epilepsy syndromes such as EAST or SeSAME [29,30]. A summary of the previous literature on potassium channels and epilepsy risk is presented in Table S1.

Voltage potassium channels are significant predictors of the clinical outcome of epilepsy. Different Kv channels may be implicated in specific epilepsy subtypes, influencing clinical outcomes such as seizure frequency and severity. In addition, understanding the phenotypic associations with Kv channel variants can guide personalized treatment strategies based on the specific channel involved [31].

Previously, we have studied the association of several sodium voltage-gated channel genes with epilepsy development and prognosis [32]. There is not enough information about the genetic contribution towards epilepsy in the Arab region. Genetic correlation with epilepsy prevalence has been tested in Arab Jordanian epilepsy patients. They were tested for polymorphisms in *CYP3A5* [33], *MTHFR* [34], *GRM4* [35], *CHRM2* [33], *SCN2A* [35], *ZNF498* [33], *SCN3B* [35], and *ABCC2* [34].

This study is part of an ongoing project on epilepsy in Saudi Arabia, focusing on its clinical and genetic aspects [32]. It aims to identify genetic variants associated with genetic epilepsy, assess their correlation with clinical phenotypes, evaluate gene–environment interactions, and determine the prevalence of specific genetic risk factors. Additionally, it seeks to enhance understanding of epilepsy’s genetic basis, supporting future pharmacogenomic drug development and improved treatment strategies.

## 2. Materials and Methods

### 2.1. Study Subjects and Design

The present study is part of an ongoing larger project to study epilepsy in Saudi Arabia, including clinical and genetic aspects of the disease [32]. The same cohort of previously published work has been used in this study, which comprised 589 Saudis of Arab descent, 296 of whom were diagnosed with epilepsy, in addition to 293 blood samples from healthy participants [32]. Epileptic patients were recruited from various healing facilities in the kingdom between November 2019 and April 2021; clinical data were obtained from their medical records. Written informed consent was obtained from all study subjects and the legal guardians of minors before this study’s commencement after they were told about the objective and aim of this study. Ethical approval was obtained for the conduct of this study (the Research Ethics Committee of King Khalid University with the approval number ECM#2019-38).

Gender and the relatively small sample size could introduce selection bias. However, potential bias due to population stratification was minimized, as the Saudi Arabian population is relatively homogeneous. Furthermore, there were no significant differences between the case and control groups regarding basic demographic characteristics. This study included patients diagnosed with epileptic seizures, including those with generalized, focal, or combined epilepsy, all of whom underwent EEG. Both refractory and non-refractory epilepsy patients were included, while individuals with non-epileptic seizures were excluded. Additional exclusion criteria included pregnancy; a history of drug abuse; the use of medications that could interfere with study outcomes; the presence of comorbid conditions that may exacerbate epilepsy; and structural epilepsy, which was ruled out through neuroimaging.

### 2.2. Polymorphism Selection, DNA Extraction, and Genotyping

When analyzing specific polymorphisms KCNAB1 and KCNJ10, we followed a systematic approach that included a review of previous studies, functional relevance, gene function, and pathway involvement. On the other hand, variants have been excluded based on criteria such as lack of association with epilepsy, low minor allele frequency, and technical limitations.

After collecting blood samples, genomic DNA was extracted directly according to the Wizard Genomic DNA Purification Kit (Promega Corporation, Madison, WI, USA). The quality and quantity of the purified DNA were then achieved by employing agarose gel electrophoresis and the Nano-Drop ND-1000 UV-vis Spectrophotometer (BioDrop, Cambridge, UK), respectively. The DNA samples were diluted with nuclease-free water to achieve a final concentration of 20 ng/μL and a final volume between 50 and 500 μL. The samples were genotyped at Gehrmann Laboratories, the University of Queensland, QLD 4072, using the Sequenom MassARRAY^®^ system (iPLEX GOLD) (Sequenom, San Diego, CA, USA) [36].

### 2.3. Statistical Analysis

All SNPs were detected to fulfill the Hardy–Weinberg equilibrium (p^2^ + 2pq + q^2^ = 1). The genotypic and allelic association was conducted using snpSTAT, 2006 Institut Català d’Oncologia software. Data description and phenotype–genotype analyses were conducted using JASP 0.16 software. For the present study, statistical significance was established at a *p*-value < 0.05. In this case–control study, both cases and controls were analyzed in the same batch.

For case–control studies, the sample size can be calculated using formulas incorporating the above factors. An example formula is as follows: n = (Z_α/2_ + Z_β_)^2^ (p_1_(1 − p_1_) + p_2_(1 − p_2_))/(p_1_ − p_2_)^2^ [37], where *n* = required sample size.

## 3. Results

### 3.1. Patient Characteristics

The data collected on the participation of epilepsy patients in the current study revealed no variation in the gender distribution between cases. Furthermore, the ages of the patients ranged from infant to 35 years in the patients. The average body mass index (BMI) was (23.2 ± 7.789). Interestingly, most of the participants were diagnosed with generalized epilepsy, and 55% of the patients had relapsed, while 79% had periodic epilepsy. We also found that 33.9% of patients had a family history of epilepsy, while 11.9% had a history of febrile seizures. The predominant seizure type was motor seizure, according to the data of this study. The general and clinical characteristics of epilepsy patients are summarized in Table 1.

### 3.2. Genetic Variants of KCN Genes

Table 2 and Table S2 describe the investigated SNPs in the genes of voltage-gated potassium channels for both cases and controls. This study included 296 patients in addition to 293 controls. The table shows the minor alleles and their frequencies, the *p*-value of HWE, in addition to the chromosomal position of the SNPs. Non-polymorphic SNPs and those not fulfilling the HWE were excluded from this study. The results of the HWE analysis show that the alleles are typically distributed among the patients, except rs16826199 of *KCNAB1* (*p*-value = 0.044) and rs7029012 of *KCNV2* (*p*-value = 0.033). Cultural understanding of the Saudi population could explain this, as consanguineous marriage is highly found in the Arab Saudi population, thus making the population nearly genetically isolated. This might have led to the abnormal distribution of the G allele of rs16826199. This trend could be seen in the controls in the A allele of rs4295133; G allele of rs2720281, rs17352408, and rs2280561; and, finally, the C allele of rs1546750 of the *KCNAB1* gene. It is also seen in the C allele of rs12402969 and the A allele of rs1186675 of the *KCNJ10* gene.

### 3.3. Genetic Association Between Genetic Variants of Voltage-Gated Potassium Channels and Epilepsy

Table 3 and Table S3 illustrate the genotypic and allelic frequencies of healthy individuals and patients, in addition to showing the *p*-values that define the correlation between each variant and epilepsy. Within *KCNAB1*, our finding asserts that both rs3755631 and rs4679773 are correlated to epilepsy; the *p*-value = 0.04 for both allelic associations. To elucidate this in detail, according to Table 3, the variant allele (G) of rs3755631 was higher among cases when compared to within controls. However, the same scenario applies to the variant allele (G) of rs4679773; therefore, we contend that these variants can influence the risk of epilepsy.

However, the genotype (A/A) of rs2820585 in the *KCNJ10* gene was slightly lower (2%) in the group of patients than among healthy individuals (2.8%). In this regard, a statistically significant association was detected for the genotypic association (*p*-value = 0.034), and therefore, we suggest that the variant A/A genotype may confer a reduced risk of epilepsy. The variant genotype A/A of rs946420 in the *KCNJ10* gene can also be designated as a protective factor against epilepsy (*p*-value = 0.045) (Table 3). In the same gene, we also inspected a compelling association between rs1186679 and epilepsy (*p*-value = 0.021). We propose that variant T/T might be involved in the development and progression of epilepsy. According to our results, rs61822012 and rs1186685 KCNJ10 were associated with epilepsy (*p*-value = 0.048 and 0.04), respectively.

### 3.4. Association of Clinical Factors of Epilepsy with Voltage-Gated K Channel Genes’ Variation

In light of the results of this study, several clinical features of epilepsy were correlated with variants of the KCN genes (Table 4, Tables S4 and S5). Age at diagnosis was associated with *KCNAB1*/rs1546750 and *KCNJ10*/rs946420, rs61822012, and rs1186685 (*p*-values = 0.045, 0.01, 0.01, and 0.01, respectively). Additionally, the findings revealed that the duration of the first seizure was related to *KCNV2*/rs10967705 and *KCNJ10*/rs11265313 (*p*-values = 0.005 and 0.002). Vitamin D was also associated with *KCNA2*/rs3887820 (*p*-value = 0.022). Furthermore, Vitamin B12 and *KCNAB1*/rs728382 were significantly correlated (*p*-value = 0.031) (Table 4).

A significant association between gender and *KCNJ10*/rs1053074 was reported (*p*-value = 0.004). Relapse was associated with *KCNAB1*/rs992353 (*p*-value = 0.008), while the history of febrile seizure was found to be related to KCNA1/rs7974459 (*p*-value = 0.027) and *KCNJ10*/rs1186689 (*p*-value = 0.020). Furthermore, potential interactions between periodic seizures and *KCNAB1*/rs4679773, *KCNJ10*/rs2820585, rs1186679, rs4656873, and rs1890532 were investigated (*p*-values = 0.029, 0.039, 0.038, 0.010, 0.015). The time to remission was correlated with *KCNJ10*/rs4656873 and rs17375748 (*p*-values = 0.049, 0.001). Furthermore, drug response was associated with *KCNAB1*/rs2280031, *KCNJ10*/rs2820585, rs946420, rs1186679, rs61822012, and rs1186685 (*p*-values = 0.042, 0.008, 0.006, 0.008, 0.004, 0.006). It was found that a family history of epilepsy was influenced by *KCNA1*/rs2227910, *KCNAB1*/rs4679773, and *KCNJ10*/rs12122979 (*p*-values = 0.039, 0.011, 0.047). Finally, epilepsy classification was correlated with *KCNV2*/rs10967728 and *KCNAB1*/rs1551066, *KCNJ9*/rs2753268 (*p*-values = 0.033, 0.020, 0.024) (Table 5).

## 4. Discussion

Epilepsy is a neurological disorder that occurs due to excessive electrical discharges in the brain that are known as seizure episodes [1]. Several essential factors affect the development and progression of the disorder; however, it is plausible that both the environment and genetics are fundamental when exploring the etiology of epilepsy [32]. The previous literature has focused on specific genes depending on their involvement in neurological pathways [38]. Voltage-gated potassium channels were comprehensively studied in relation to the risk of epilepsy, and many polymorphisms have been identified as biomarkers involved in epilepsy [39].

Voltage-gated potassium channels are critical in epilepsy genetics by influencing neuronal excitability, seizure susceptibility, and treatment responses. Their genetic mutations can lead to significant alterations in neuronal function, contributing to the development and progression of epilepsy. Key points highlight their specific role, including the Regulation of Neuronal Membrane Potential by Repolarization, where Kv channels facilitate the repolarization phase of action potentials. In addition, an Impact on Excitability occurs when a dysfunction in Kv channels can lead to prolonged depolarization and increased neuronal excitability, leading to the hyperexcitability characteristic of epilepsy [40].

In this study, we revealed the association between different genetic variants of voltage-gated potassium channels and epilepsy risk, and, accordingly, we suggest that both rs3755631 and rs4679773 of *KCNAB1* are correlated with epilepsy. We also contend that the variant alleles (G) of both polymorphisms can influence the risk of epilepsy. Other studies support the fact that *KCNAB1* is a vulnerable gene for lateral temporal epilepsy, causing it to alter susceptibility to focal epilepsy [41,42]. We also that the A/A variant genotype of rs2820585 in *KCNJ10* may confer a reduced risk of epilepsy, as well as the A/A genotype of rs946420 in *KCNJ10*. Furthermore, a compelling association between rs1186679 and epilepsy was detected, and we propose that the variant T/T could be involved in the development and progression of epilepsy. Based on our results, we infer that both rs61822012 and rs1186685 *KCNJ10* are involved in the risk of epilepsy. However, in contrast to our findings, rs1186685 and rs12122979 were not associated with the focal epilepsy susceptibility in the Chinese Han population [43]. In a study made in Jordan, it was shown that genes related to the potassium ion channel protein are genetically associated with the diagnosis of epileptic seizures in patients [44].

Furthermore, in the current study, we investigated the association of the clinical factors of epilepsy with the voltage-gated potassium channel gene variant. It is intelligible that definite genotypes can contribute to variations in epilepsy phenotypes. Age at diagnosis was correlated with *KCNAB* and *KCNJ10*. We also found that *KCNV2* and *KCNJ10* can interfere with the duration of the first seizure. The levels of Vitamin D and Vitamin B12 levels among epilepsy patients were influenced by *KCNA2* and *KCNAB1*, respectively. Vitamin D is significant as a prospective intervention for epilepsy, and it has been declared that among epilepsy patients, the level of vitamin D is low [45,46]. In this regard, detecting the genetic variant of voltage-gated potassium channel genes is fundamental as it may manipulate the level of such essential vitamins.

The incidence of epilepsy is slightly higher in men than in women [47], and according to our results, gender and the *KCNJ10* variant were correlated. We assert that genetic variants of *KCN* genes may impact the prevalence of epilepsy among men and women. Genetic variants can be used as predictive factors for the recurrence of epilepsy, and in light of our findings, *KCNAB1* was found to impact relapse in epilepsy. KCNA1 influenced the history of febrile seizures and the history of epilepsy. Our findings also infer that *KCNAB1* and *KCNJ10* may enhance periodic spasms. Genetic variation can modulate the response to medications. In this study, we implied that *KCNJ10* and *KCNAB1* may mediate drug responsiveness in epilepsy patients. Furthermore, the time to remission was correlated with *KCNJ10*, and *KCNV2* and *KCNJ9* influenced the classification of epilepsy. However, we cannot generalize the findings of this study simply due to the population’s ethnicity divergence.

Over the last few years, knowledge awareness and cultural understanding about epilepsy diagnosis have increased [48]. This has been reflected through more cultural acceptance of epilepsy patients in different aspects of life [49]. However, there remains a significant gap in knowledge regarding the genetic associations of epilepsy in the Arab region. This lack of information hinders the development of personalized pharmacogenomic treatment options for epilepsy in this population. The results of the current study may contribute to bridging this gap, as they shed light on the genetic associations of epilepsy in a Saudi Arabian population, which may have genetically distinct sub-populations.

Phenotype–genotype studies are essential for identifying correlations between genetic markers and epilepsy, providing a deeper understanding of its prevalence and clinical manifestations. These insights could pave the way for more targeted treatment strategies, improving both the diagnosis and management of epilepsy in the region. By incorporating genetic information into clinical practice, it may be possible to tailor treatments based on individual genetic profiles, allowing for more effective and personalized care. Furthermore, the development of pharmacogenomic drugs specifically tailored to the genetic landscape of this population could provide better outcomes than the currently available treatments, which often take a more generalized approach.

To better understand the pathogenic mechanisms underlying epilepsy in this population, experimental studies are needed to demonstrate the functional consequences of the genetic variants identified in this study. These studies could involve in vitro or in vivo models to examine how these genetic factors contribute to seizure susceptibility and the efficacy of potential treatments.

## 5. Conclusions

This study highlights a potential link between voltage-gated potassium channels and the risk of epilepsy, especially concerning the genetic variations in the *KCNAB1* and *KCNJ10* genes. Our findings suggest that variations in *KCN* genes may affect the clinical presentation and prognosis of epilepsy. Further genotype–phenotype studies are needed to enhance diagnostic accuracy and optimize treatment strategies. Additional research on the genetic basis of epileptic seizures will be essential for advancing pharmacogenomic approaches and expanding treatment options for patients.

## Figures and Tables

**Table 1:** General and clinical characteristics of the epilepsy patients.

General/Clinical Characteristics		Mean/Frequency
Age at diagnosis		9.7 ± 7.986
BMI		23.2 ± 7.789
Duration of first seizure (sec)		8.703 ± 14.032
Gender	Male	51%
	Female	49%
Time to remission	No	28.5%
	Yes (seizure-free immediately)	52.3%
	Yes (seizure-free within 6 months)	18.7%
Epilepsy classification	Focal (partial) onset	16%
	Generalized onset	76%
	Combined generalized and focal onset	8%
Seizure classification	Motor	94.6%
	Non-motor	5.4%
History of febrile seizure	Yes	11.9%
	No	88.1%
Family history of epilepsy	Yes	33.9%
	No	66.1%
AED drug	Yes	6%
	No	94%
First drug responsiveness	Yes	64%
	No	36%
Relapse	Yes	45%
	No	55%
Periodic epilepsy	Yes	21%
	No	79%

**Table 2:** Description of KCN gene variants among epilepsy cases and healthy controls.

Gene	SNP ID	SNP Position ^a^	Cases (n = 296)			Controls (n =293)	
			MA ^b^	MAF ^c^	HWE ^d^ *p*-Value	MAF ^c^	HWE ^d^ *p*-Value
*KCNV2*	rs7029012	9:2717698	C	0.36	0.033	0.40	0.18
*KCNAB1*	rs4295133	3:156516479	A	0.47	0.48	0.49	0.003
	rs2720281	3:156512429	G	0.47	0.10	0.44	0.012
	rs17352408	3:156484880	G	0.28	0.19	0.29	0.032
	rs1546750	3:156468103	C	0.48	0.062	0.47	6 × 10^−4^
	rs2280561	3:156460417	G	0.44	1.00	0.47	1 × 10^−3^
	rs16826199	3:156452654	G	0.08	0.044	0.05	0.57
*KCNJ10*	rs12402969	1:160050447	C	0.01	1.00	0.01	0.017
	rs1186675	1:160069294	A	0.02	0.11	0.03	0.019

^a^ Chromosome positions are based on NCBI Human Genome Assembly Build. ^b^ MA: minor allele. ^c^ MAF: minor allele frequency. ^d^ HWE: Hardy—Weinberg equilibrium.

**Table 3:** Genetic association between KCN polymorphisms and epilepsy.

Gene	SNP ID	Allelic and Genotypic Frequencies in Cases and Controls			
		Allele/Genotype	Cases (n = 495)	Controls (n = 497)	*p*-Value *
*KCNAB1*	rs3755631	C	539 (91%)	550 (94%	0.04
		G	53 (0.09%)	34 (0.06%)	
	rs4679773	C	300 (51%)	330 (57%)	0.04
		G	288 (49%)	250 (43%)	
*KCNJ10*	rs2820585	G/G	204 (68.9%)	225 (77.3%)	0.034
		A/G	86 (29.1%)	58 (19.9%)	
		A/A	6 (2%)	8 (2.8%)	
	rs946420	C/C	204 (69.2%)	223 (77.2%)	0.045
		C/A	85 (28.8%)	58 (20.1%)	
		A/A	6 (2%)	8 (2.8%)	
	rs1186679	C/C	203 (68.8%)	226 (77.4%)	0.021
		C/T	87 (29.5%)	58 (19.9%)	
		T/T	5 (1.7%)	8 (2.7%)	
	rs61822012	A/A	203 (69.3%)	226 (77.7%)	0.048
		A/G	84 (28.7%)	58 (19.9%)	
		G/G	6 (2%)	7 (2.4%)	
	rs1186685	A	495 (84%)	513 (88%)	0.04
		G	97 (17%)	71 (13%)	
	rs12122979	A/A	205 (69.3%)	229 (78.4%)	0.018
		G/A	85 (28.7%)	55 (18.8%)	
		G/G	6 (2%)	8 (2.7%)	

* *p* < 0.05 is considered significant.

**Table 4:** Regression analysis of KCN variants in relation to epilepsy traits.

Gene	SNP ID	Age at Diagnosis	Duration of First Seizure	BMI	VIT.D	VIT B12
*KCNA2*	rs3887820	0.832	0.265	0.133	0.022	0.856
*KCNV2*	rs10967705	0.510	0.005	0.784	0.794	0.742
	rs1546750	0.045	0.776	0.249	0.904	0.320
	rs728382	0.866	0.069	0.244	0.326	0.031
	rs946420	0.01	0.189	0.146	0.093	0.469
	rs11265313	0.320	0.002	0.573	0.172	0.778
	rs61822012	0.01	0.330	0.118	0.093	0.469
	rs1186685	0.01	0.189	0.146	0.146	0.469

Linear regression using ANOVA test. *p*-value <0.05 is significant.

**Table 5:** Regression analysis exploring the association between KCN variants and epilepsy features.

Gene	SNP ID	Gender	Relapse	History of Febrile Seizure	Periodic Seizure	Time to Remission	Drug Responsiveness	Drug Level	Family History of Epilepsy	Epilepsy Classification	Seizure Classification
*KCNA1*	rs2227910	0.115	0.316	0.440	0.531	0.803	0.768	0.142	0.039	0.825	0.882
	rs7974459	0.372	0.572	0.027	0.191	0.176	0.525	0.815	0.544	0.331	0.954
*KCNV2*	rs10967728	0.711	0.209	0.327	0.365	0.735	0.544	0.462	0.556	0.033	0.281
*KCNA-B1*	rs992353	0.787	0.008	0.934	0.508	0.355	0.724	0.466	0.376	0.131	0.730
	rs4679773	0.167	0.732	0.324	0.029	0.392	0.370	0.291	0.011	0.331	0.500
	rs1551066	0.665	0.750	0.599	0.175	0.513	0.207	0.678	0.093	0.020	0.997
	rs2280031	0.723	0.715	0.683	0.782	0.254	0.042	0.522	0.851	0.954	0.456
*KCNJ10*	rs1053074	0.004	0.486	0.409	0.086	0.134	0.791	0.128	0.545	0.647	0.606
	rs2820585	0.203	0.787	0.321	0.039	0.643	0.008	0.279	0.448	0.652	0.381
	rs946420	0.176	0.741	0.205	0.153	0.781	0.006	0.209	0.403	0.896	0.297
	rs1186679	0.188	0.752	0.329	0.038	0.612	0.008	0.284	0.482	0.648	0.387
	rs4656873	0.440	0.798	0.313	0.010	0.049	0.879	0.082	0.947	0.482	0.786
	rs17375748	0.270	0.399	0.193	0.542	0.001	0.659	0.729	0.140	0.616	0.208
	rs61822012	0.217	0.651	0.197	0.161	0.669	0.004	0.203	0.425	0.888	0.290
	rs1890532	0.665	0.693	0.398	0.015	0.070	0.893	0.137	0.540	0.585	0.890
	rs1186689	0.324	0.984	0.020	0.536	0.417	0.084	0.072	1.000	0.712	0.436
	rs1186685	0.176	0.741	0.205	0.153	0.781	0.006	0.209	0.403	0.896	0.297
	rs12122979	0.251	0.225	0.437	0.512	0.591	0.056	0.372	0.047	0.902	0.629
*KCNJ9*	rs2753268	0.509	0.238	0.213	0.368	0.231	0.429	0.437	0.134	0.024	0.172

Linear regression using Pearson’s r test. *p*-value < 0.05 is significant.

## Data Availability

The datasets generated and/or analyzed during this study are available from the corresponding author upon reasonable request.

## Supplementary Materials

The following supporting information can be downloaded at https://www.mdpi.com/article/10.3390/medicina61030396/s1, Table S1: Summary of the Previous Literature on Potassium Channels and Epilepsy Risk, Table S2: Description of KCN gene variants among epilepsy cases and healthy controls, Table S3: Genetic association between the KCN polymorphisms and epilepsy, Table S4: Regression analysis of KCN variants and epilepsy features, Table S5: Regression analysis of KCN variants and epilepsy features.
