# Supplementary material for: Association of Voltage-Gated Potassium Channel Polymorphisms with the Risk and Prognosis of Epilepsy in the Saudi Population: A Case–Control Study
Source: Medicina (Kaunas). 2025 Feb 25;61(3):396. doi: 10.3390/medicina61030396 (PMC11943637; doi:10.3390/medicina61030396)
Supplement: Supplementary file 1 [file medicina-61-00396-s001.zip › Supplementary Table S1.pdf]

Table S1. Summary of Previous Literature on Potassium Channels and Epilepsy Risk.

Gene	Chromosome Location	Encoded Protein	Epilepsy Phenotypes	Mutation Effects	References
**KCNA1**	Chr 12	Kv1.1 (Voltage-gated K^+ channel)	Generalized or partial seizures, episodic ataxia, neonatal epileptic encephalopathy (NEE)	Loss of function mutations reduce K^+ currents	[17-21]
**KCNA2**	Chr 1	Kv1.2 (Voltage-gated K^+ channel)	Epileptic encephalopathy (EE), ataxia, myoclonic epilepsy, BFNIS, GEFS+, Dravet syndrome	Some mutations cause loss of function (e.g., p.Ile263Thr), others cause gain of function (e.g., p.Arg297Gln)	[22-25]
**KCNV2**	Chr 9	Kv8.2 (Regulatory subunit)	Febrile seizures, epileptic encephalopathy, severe refractory epilepsy	p.Arg7Lys decreases K^+ current, p.Met285Arg linked to severe epilepsy	[26]
**KCNAB2**	Chr 1	Kvβ2 (Regulatory subunit)	Severe epilepsy	Hemizygous deletion causes loss of function	[27]
**KCNJ10**	Chr 1	Kir4.1 (Inward rectifier K^+ channel)	EAST syndrome, SeSAME syndrome, seizure susceptibility	p.Arg271Cys linked to seizures, p.Arg65Cys causes loss of function	[28-30]
