# Supplementary material for: Association of Voltage-Gated Potassium Channel Polymorphisms with the Risk and Prognosis of Epilepsy in the Saudi Population: A Case–Control Study
Source: Medicina (Kaunas). 2025 Feb 25;61(3):396. doi: 10.3390/medicina61030396 (PMC11943637; doi:10.3390/medicina61030396)
Supplement: Supplementary file 1 [file medicina-61-00396-s001.zip › Supplementary Table S2.pdf]

Table S2. Description of KCN genes variants among epilepsy cases and healthy controls.

Gene	SNP ID	SNP position[a]	Cases (n =296)			Controls (n =293)	
			MA[b]	MAF[c]	HWE[d]P-value	MAF[c]	HWE[d]P-value
KCNA1	rs2227910	12:4912182	G	0.31	0.1	0.27	0.66
	rs112561866	12:4912725	A	0.00	N/A	0.00	N/A
	rs7974459	12:4914547	T	0.32	0.14	0.29	1.00
KCNA2	rs3887820	1:110605458	A	0.09	0.3	0.08	1.00
	rs786205232	1:110603893	A	0.00	N/A	0.00	N/A
KCNV2	rs7029012	9:2717698	C	0.36	**0.033**	0.40	0.18
	rs10967705	9:2717922	C	0.39	0.069	0.44	0.29
	rs10967728	9:2721794	C	0.47	0.56	0.46	0.48
KCNAB1	rs992353	3:156538672	C	0.48	0.73	0.49	0.29
	rs4295133	3:156516479	A	0.47	0.48	0.49	**0.003**
	rs2280299	3:156524176	G	0.4	0.18	0.41	0.27
	rs2720281	3:156512429	G	0.47	0.10	0.44	**0.012**
	rs17352408	3:156484880	G	0.28	0.19	0.29	**0.032**
	rs1546750	3:156468103	C	0.48	0.062	0.47	**6e-4**
	rs2280561	3:156460417	G	0.44	1.00	0.47	**1e-3**
	rs3755631	3:156454654	G	0.09	0.07	0.06	1.00
	rs4679773	3:156435979	G	0.49	1.00	0.43	0.81
	rs728382	3:156238980	G	0.33	0.29	0.34	0.24
	rs9816126	3:156147466	T	0.17	0.053	0.16	0.078
	rs1386956	3:156087834	A	0.35	0.37	0.36	0.90
	rs16826199	3:156452654	G	0.08	**0.044**	0.05	0.57
	rs1551066	3:156292136	T	0.46	0.91	0.46	0.56
	rs2280031	3:156531425	C	0.14	0.63	0.18	0.69
KCNJ10	rs1053074	1:160039331	A	0.48	0.56	0.47	0.10
	rs2820585	1:160069869	A	0.17	0.53	0.13	0.11
	rs946420	1:160069627	A	0.16	0.53	0.13	0.11
	rs1186679	1:160063761	T	0.16	0.29	0.13	0.11
	rs7512587	1:160063353	T	0.38	0.80	0.38	0.26
	rs4656873	1:160061540	C	0.12	0.28	0.15	0.81
	rs11265313	1:160059666	A	0.46	0.48	0.43	0.55
	rs1186689	1:160053263	T	0.49	0.73	0.49	0.81
	rs17375748	1:160040361	T	0.08	0.45	0.06	0.32
	rs61822012	1:160071368	G	0.16	0.53	0.12	0.17
	rs2486253	1:160039629	A	0.2	1.00	0.2	1.00
	rs1130183	1:160041722	A	0.00	1.00	0.01	1.00
	rs1186688	1:160055093	C	0.38	0.90	0.38	0.21
	rs3795339	1:160041721	T	0.00	N/A	0.00	N/A
	rs12729701	1:160042616	G	0.17	0.31	0.15	0.36
	rs12402969	1:160050447	C	**0.01**	1.00	0.01	**0.017**
	rs1890532	1:160064785	G	0.13	0.19	0.16	1.00
	rs1186675	1:160069294	A	**0.02**	0.11	0.03	**0.019**
	rs6690889	1:160063712	C	0.41	0.72	0.41	0.33
	rs1186685	1:160059422	G	0.16	0.53	0.12	0.052
	rs12122979	1:160053302	G	0.47	0.73	0.45	0.34
KCNJ9	rs6677510	1:160082329	G	0.43	0.41	0.42	0.071
	rs11265317	1:160083552	A	0.01	1.00	0.02	0.075
	rs2737702	1:160085315	T	0.38	0.39	0.4	0.54

	rs2737703	1:160086142	T	0.38	0.22	0.38	0.32
	rs2753268	1:160088462	A	0.12	0.28	0.11	0.76
	rs2494211	1:160088822	T	0.36	0.13	0.37	0.53

[a]Chromosome positions are based on NCBI Human Genome Assembly Build.[b]MA: minor allele. [c]MAF: minor allele frequency. [d]HWE: Hardy—Weinberg equilibrium. N/A: not applicable
