# Supplementary material for: Association of Voltage-Gated Potassium Channel Polymorphisms with the Risk and Prognosis of Epilepsy in the Saudi Population: A Case–Control Study
Source: Medicina (Kaunas). 2025 Feb 25;61(3):396. doi: 10.3390/medicina61030396 (PMC11943637; doi:10.3390/medicina61030396)
Supplement: Supplementary file 1 [file medicina-61-00396-s001.zip › Supplementary Table S3.pdf]

Table S3. Genetic association between the KCN polymorphisms and epilepsy.

Gene	SNP ID	Allelic and Genotypic Frequencies in Cases and Controls			
		**Allele/Genotype**	**Cases (n =495)**	**Controls (n =497)**	**P-value***
KCNA1	rs2227910	C	424 (69%)	406 (73%)	0.15
		G	160 (31%)	184 (27%)	
		C/C	146 (49.5%)	152 (52%)	0.11
		G/C	114 (38.6%)	120 (41.1%)	
		G/G	35 (11.9%)	20 (6.8%)	
	rs7974459	C	403 (68%)	415 (71%)	0.22
		T	189 (32%)	167 (29%)	
		C/C	143 (48.3%)	148 (50.9%)	0.29
		T/C	117 (39.5%)	119 (40.9%)	
		T/T	36 (12.2%)	24 (8.2%)	
KCNA2	rs3887820	C	537 (91%)	534 (92)	0.52
		A	55 (9%)	48 (8%)	
		C/C	245 (82.8%)	245 (84.2%)	0.69
		C/A	47 (15.9%)	44 (15.1%)	
		A/A	4 (1.4%)	2 (0.7%)	
KCNV2	rs10967705	G	358 (61%)	324 (56%)	0.09
		C	232 (39%)	256 (44%)	
		G/G	116 (39.3%)	95 (32.8%)	0.24
		C/G	126 (42.7%)	134 (46.2%)	
		C/C	53 (18%)	61 (21%)	
	rs10967728	G	313 (53%)	312 (54%)	0.84
		C	277 (47%)	270 (46%)	
		G/G	80 (27.1%)	87 (29.9%)	0.56
		G/C	153 (51.9%)	138 (47.4%)	
		C/C	62 (21%)	66 (22.7%)	
KCNAB1	rs992353	T	304 (52%)	294 (51%)	0.72
		C	286 (48%)	288(49%)	
		T/T	80 (27.1%)	79 (27.1%)	0.83
		T/C	144 (48.8%)	136 (46.7%)	
		C/C	71 (24.1%)	76 (26.1%)	
	rs2280299	A	346 (60%)	343 (59%)	0.80
		G	232 (40%)	237 (41%)	
		A/A	109 (37.7%)	106 (36.5%)	0.96
		G/A	128 (44.3%)	131 (45.2%)	
		G/G	52 (18%)	53 (18.3%)	
	rs1546750	A	309 (52%)	305 (53%)	0.89
		C	283 (48%)	275 (47%)	
		A/A	89 (30.1%)	95 (32.8%)	0.53
		C/A	131 (44.3%)	115 (39.7%)	

		C/C	76 (25.7%)	80 (27.6%)	
	rs3755631	C	539 (91%)	550 (94%	**0.04**
		G	53 (0.09%)	34 (0.06%)	
		C/C	248 (83.8%)	259 (88.7%)	0.093
		C/G	43 (14.5%)	32 (11%)	
		G/G	5 (1.7%)	1 (0.3%)	
	rs4679773	C	300 (51%)	330 (57%)	**0.04**
		G	288 (49%)	250 (43%)	
		C/C	76 (25.9%)	95 (32.8%)	0.13
		G/C	148 (50.3%)	140 (48.3%)	
		G/G	70 (23.8%)	55 (19%)	
	rs728382	A	385 (67%)	381(66%)	0.67
		G	191(33%)	199(34%)	
		A/A	133 (46.2%)	130 (44.8%)	0.92
		G/A	119 (41.3%)	121 (41.7%)	
		G/G	36 (12.5%)	39 (13.4%)	
	rs9816126	G	480 (83%)	483 (84%)	0.81
		T	96 (17%)	93 (16%)	
		G/G	205 (71.2%)	207 (71.9%)	0.97
		G/T	70 (24.3%)	69 (24%)	
		T/T	13 (4.5%)	12 (4.2%)	
	rs1386956	G	385 (65%)	371 (64%)	0.58
		A	205 (35%)	211 (36%)	
		G/G	129 (43.7%)	119 (40.9%)	0.77
		G/A	127 (43%)	133 (45.7%)	
		A/A	39 (13.2%)	39 (13.4%)	
	rs1551066	C	319 (54%)	318 (54%)	0.89
		T	271 (46%)	266 (46%)	
		C/C	87 (29.5%)	89 (30.5%)	0.95
		T/C	145 (49.1%)	140 (48%)	
		T/T	63 (21.4%)	63 (21.6%)	
	rs2280031	T	507 (86%)	478 (82%)	0.06
		C	83(14%)	106 (18%)	
		T/T	219 (74.2%)	194 (66.4%)	0.11
		C/T	69 (23.4%)	90 (30.8%)	
		C/C	7 (2.4%)	8 (2.7%)	
KCNJ10	rs1053074	C	309 (52%)	310 (53%)	0.66
		A	283 (48%)	270 (47%)	
		C/C	78 (26.4%)	90 (31%)	0.24
		C/A	153 (51.7%)	130 (44.8%)	
		A/A	65 (22%)	70 (24.1%)	
	rs2820585	G	494 (83%)	508 (87%)	0.06
		A	98 (17%)	74 (13%)	
		G/G	204 (68.9%)	225 (77.3%)	**0.034**

		A/G	86 (29.1%)	58 (19.9%)	
		A/A	6 (2%)	8 (2.8%)	
	rs946420	C	493 (84%)	504 (87%)	0.07
		A	97 (16%)	74 (13%)	
		C/C	204 (69.2%)	223 (77.2%)	**0.045**
		C/A	85 (28.8%)	58 (20.1%)	
		A/A	6 (2%)	8 (2.8%)	
	rs1186679	C	493 (84%)	510 (87%)	0.06
		T	97 (16%)	74 (13%)	
		C/C	203 (68.8%)	226 (77.4%)	**0.021**
		C/T	87 (29.5%)	58 (19.9%)	
		T/T	5 (1.7%)	8 (2.7%)	
	rs7512587	C	368 (62%)	348 (62%)	0.81
		T	222 (38%)	216 (38%)	
		C/C	116 (39.3%)	112 (39.7%)	0.81
		C/T	136 (46.1%)	124 (44%)	
		T/T	43 (14.6%)	46 (16.3%)	
	rs4656873	T	517 (88%)	496 (85%)	0.17
		C	71 (12%)	86 (15%)	
		T/T	225 (76.5%)	212 (72.8%)	0.17
		T/C	67 (22.8%)	72 (24.7%)	
		C/C	2 (0.7%)	7 (2.4%)	
	rs11265313	G	319 (54%)	329 (57%)	0.29
		A	271 (46%)	247 (43%)	
		G/G	83 (28.1%)	91 (31.6%)	0.56
		G/A	153 (51.9%)	147 (51%)	
		A/A	59 (20%)	50 (17.4%)	
	rs1186689	G	295 (51%)	287 (51%)	N/A
		T	289 (49%)	281 (49%)	
		G/G	76 (26%)	71 (25%)	0.88
		G/T	143 (49%)	145 (51.1%)	
		T/T	73 (25%)	68 (23.9%)	
	rs17375748	C	542 (92%)	547 (94%)	0.16
		T	50 (0.08%)	37 (06%)	
		C/C	249 (84.1%)	257 (88%)	0.39
		C/T	44 (14.9%)	33 (11.3%)	
		T/T	3 (1%)	2 (0.7%)	
	rs61822012	A	490 (84%)	510 (88%)	0.05
		G	96 (16%)	72 (12%)	
		A/A	203 (69.3%)	226 (77.7%)	**0.048**
		A/G	84 (28.7%)	58 (19.9%)	
		G/G	6 (2%)	7 (2.4%)	
	rs2486253	C	475 (80%)	466 (80%)	0.94
		A	117 (20%)	116 (20%)	

		C/C	190 (64.2%)	186 (63.9%)	1.00
		C/A	95 (32.1%)	94 (32.3%)	
		A/A	11 (3.7%)	11 (3.8%)	
	rs1130183	G	591 (100%)	581 (99%)	0.31
		A	1 (0%)	3 (1%)	
		G/G	295 (99.7%)	289 (99%)	0.30
		A/G	1 (0.3%)	3 (1%)	
	rs1186688	T	367 (62%)	357 (62%)	1.00
		C	225 (38%)	219 (38%)	
		T/T	113 (38.2%)	116 (40.3%)	0.56
		C/T	141 (47.6%)	125 (43.4%)	
		C/C	42 (14.2%)	47 (16.3%)	
	rs12729701	A	489 (83%)	495 (85%)	0.32
		G	103 (17%)	89 (15%)	
		A/A	199 (67.2%)	212 (72.6%)	0.18
		A/G	91 (30.7%)	71 (24.3%)	
		G/G	6 (2%)	9 (3.1%)	
	rs1890532	C	515 (87%)	491 (84%)	0.15
		G	77 (14%)	93 (17%)	
		C/C	221 (74.7%)	206 (70.5%)	0.16
		C/G	73 (24.7%)	79 (27.1%)	
		G/G	2 (0.7%)	7 (2.4%)	
	rs6690889	T	350 (59%)	340 (59%)	0.86
		C	240 (41%)	238 (41%)	
		T/T	102 (34.6%)	104 (36%)	0.60
		C/T	146 (49.5%)	132 (45.7%)	
		C/C	47 (15.9%)	53 (18.3%)	
	rs1186685	A	495 (84%)	513 (88%)	**0.04**
		G	97 (17%)	71 (13%)	
		A/A	205 (69.3%)	229 (78.4%)	**0.018**
		G/A	85 (28.7%)	55 (18.8%)	
		G/G	6 (2%)	8 (2.7%)	
	rs12122979	A	313 (53%)	317 (55%)	0.49
		G	277 (48%)	259 (45%)	
		A/A	81 (27.5%)	83 (28.8%)	0.73
		G/A	151 (51.2%)	151 (52.4%)	
		G/G	63 (21.4%)	54 (18.8%)	
KCNJ9	rs6677510	A	339 (57%)	336 (58%)	0.81
		G	253 (43%)	244 (42%)	
		A/A	93 (31.4%)	105 (36.2%)	0.13
		G/A	153 (51.7%)	126 (43.5%)	
		G/G	50 (16.9%)	59 (20.3%)	
	rs11265317	C	586 (99%)	574 (98%)	0.30
		A	6 (1%)	10 (2%)	

		C/C	290 (98%)	283 (96.9%)	0.42
		C/A	6 (2%)	8 (2.7%)	
		A/A	0 (0%)	1 (0.3%)	
	rs2737702	A	367 (62%)	347 (60%)	0.54
		T	225 (38%)	229 (40%)	
		A/A	110 (37.2%)	107 (37.1%)	0.46
		T/A	147 (49.7%)	133 (46.2%)	
		T/T	39 (13.2%)	48 (16.7%)	
	rs2737703	C	365 (62%)	358 (62%)	0.92
		T	227 (38%)	220 (38%)	
		C/C	107 (36.1%)	115 (39.8%)	0.24
		T/C	151 (51%)	128 (44.3%)	
		T/T	38 (12.8%)	46 (15.9%)	
	rs2753268	G	520 (88%)	519 (89%)	0.47
		A	72 (12%)	63 (11%)	
		G/G	226 (76.3%)	232 (79.7%)	0.35
		A/G	68 (23%)	55 (18.9%)	
		A/A	2 (0.7%)	4 (1.4%)	
	rs2494211	C	379 (64%)	368 (63%)	0.77
		T	213 (36%)	214 (37%)	
		C/C	115 (38.9%)	119 (40.9%)	0.26
		T/C	149 (50.3%)	130 (44.7%)	
		T/T	32 (10.8%)	42 (14.4%)	

*P-Value <0.05 considered as significant. N/A not applicable
