# Supplementary material for: Association of Voltage-Gated Potassium Channel Polymorphisms with the Risk and Prognosis of Epilepsy in the Saudi Population: A Case–Control Study
Source: Medicina (Kaunas). 2025 Feb 25;61(3):396. doi: 10.3390/medicina61030396 (PMC11943637; doi:10.3390/medicina61030396)
Supplement: Supplementary file 1 [file medicina-61-00396-s001.zip › Supplementary Table S4.pdf]

Table S4. Regression analysis of KCN variants and epilepsy features.

Gene	SNP ID	Age at diagnosis	Duration of first seizure	BMI	VIT.D	VIT B12
KCNA1	rs2227910	0.862	0.244	0.430	0.445	0.508
	rs7974459	0.543	0.417	0.238	0.943	0.697
KCNA2	rs3887820	0.832	0.265	0.133	**0.022**	0.856
KCNV2	rs10967705	0.510	**0.005**	0.784	0.794	0.742
	rs10967728	0.937	0.082	0.216	0.843	0.916
KCNAB1	rs992353	0.539	0.908	0.613	0.627	0.341
	rs2280299	0.911	0.627	0.738	0.584	0.743
	rs1546750	**0.045**	0.776	0.249	0.904	0.320
	rs3755631	0.412	0.512	0.148	N/A	N/A
	rs4679773	0.314	0.504	0.106	0.836	0.858
	rs728382	0.866	0.069	0.244	0.326	**0.031**
	rs9816126	0.330	0.388	0.942	0.269	0.890
	rs1386956	0.515	0.808	0.160	0.424	0.056
	rs1551066	0.715	0.940	0.332	0.223	0.488
	rs2280031	0.264	0.874	0.440	0.168	0.550
KCNJ10	rs1053074	0.278	0.316	0.386	0.344	0.149
	rs2820585	0.074	0.256	0.176	0.171	0.753
	rs946420	**0.01**	0.189	0.146	0.093	0.469
	rs1186679	0.069	0.256	0.185	0.171	0.753
	rs7512587	0.572	0.514	0.611	0.617	0.942
	rs4656873	0.747	0.165	0.838	0.963	0.900
	rs11265313	0.320	**0.002**	0.573	0.172	0.778
	rs1186689	0.697	0.442	0.259	0.308	0.778
	rs17375748	0.993	0.810	0.247	0.499	0.357
	rs61822012	**0.01**	0.330	0.118	0.093	0.469
	rs2486253	0.268	0.391	0.457	0.259	0.094
	rs1186688	0.395	0.199	0.761	0.070	0.904
	rs12729701	0.691	0.654	0.561	0.429	0.549
	rs1890532	0.772	0.206	0.495	0.956	0.779
	rs1186689	0.362	0.359	0.363	0.054	0.904
	rs1186685	**0.01**	0.189	0.146	0.146	0.469
	rs12122979	0.686	0.813	0.616	0.656	0.475
KCNJ9	rs6677510	0.900	0.788	0.986	0.839	0.923
	rs2737702	0.714	0.799	0.557	0.665	0.828
	rs2737703	0.869	0.698	0.270	0.721	0.479
	rs2753268	0.103	0.770	0.719	0.302	0.458
	rs2494211	0.668	0.915	0.520	0.562	0.828

Linear regression using ANOVA test. P value <0.05 is significant (shown in bold)
