# Supplementary material for: Association of Voltage-Gated Potassium Channel Polymorphisms with the Risk and Prognosis of Epilepsy in the Saudi Population: A Case–Control Study
Source: Medicina (Kaunas). 2025 Feb 25;61(3):396. doi: 10.3390/medicina61030396 (PMC11943637; doi:10.3390/medicina61030396)
Supplement: Supplementary file 1 [file medicina-61-00396-s001.zip › Supplementary Table S5.pdf]

Table S5. Regression analysis of KCN variants and epilepsy features.

Gene	SNP ID	Gender	Relapse	History of febrile seizure	Periodic Seizure	Time to remission	Drug responsiveness	Drug level	Family history of epilepsy	Epilepsy classification	Seizure classification
KCNA1	rs2227910	0.115	0.316	0.440	0.531	0.803	0.768	0.142	**0.039**	0.825	0.882
	rs7974459	0.372	0.572	**0.027**	0.191	0.176	0.525	0.815	0.544	0.331	0.954
KCNA2	rs3887820	0.547	0.341	0.914	0.416	0.276	0.345	0.317	0.569	0.422	0.408
KCNV2	rs10967705	0.838	0.242	0.073	0.643	0.111	0.588	0.574	0.604	0.638	0.662
	rs10967728	0.711	0.209	0.327	0.365	0.735	0.544	0.462	0.556	**0.033**	0.281
KCNAB1	rs992353	0.787	**0.008**	0.934	0.508	0.355	0.724	0.466	0.376	0.131	0.730
	rs2280299	0.143	0.934	0.979	0.627	0.777	0.241	0.855	0.291	0.800	0.880
	rs1546750	0.291	0.327	0.886	0.513	0.341	0.928	0.904	0.609	0.946	0.955
	rs3755631	0.612	0.851	0.558	0.311	0.708	0.233	0.559	0.109	0.597	0.149
	rs4679773	0.167	0.732	0.324	**0.029**	0.392	0.370	0.291	**0.011**	0.331	0.500
	rs728382	0.868	0.385	0.053	0.841	0.059	0.715	0.487	0.587	0.408	0.105
	rs9816126	0.653	0.692	0.954	0.915	0.131	0.171	0.066	0.238	0.344	0.217
	rs1386956	0.960	0.476	0.645	0.935	0.248	0.995	0.437	0.193	0.406	0.553
	rs1551066	0.665	0.750	0.599	0.175	0.513	0.207	0.678	0.093	**0.020**	0.997
	rs2280031	0.723	0.715	0.683	0.782	0.254	**0.042**	0.522	0.851	0.954	0.456
KCNJ10	rs1053074	**0.004**	0.486	0.409	0.086	0.134	0.791	0.128	0.545	0.647	0.606
	rs2820585	0.203	0.787	0.321	**0.039**	0.643	**0.008**	0.279	0.448	0.652	0.381
	rs946420	0.176	0.741	0.205	0.153	0.781	**0.006**	0.209	0.403	0.896	0.297
	rs1186679	0.188	0.752	0.329	**0.038**	0.612	**0.008**	0.284	0.482	0.648	0.387
	rs7512587	0.349	0.392	0.232	0.536	0.262	0.114	0.066	0.613	0.557	0.778
	rs4656873	0.440	0.798	0.313	**0.010**	**0.049**	0.879	0.082	0.947	0.482	0.786
	rs11265313	0.171	0.561	0.730	0.354	0.616	0.101	0.242	0.729	0.511	0.633
	rs1186689	0.408	0.855	0.761	0.246	0.834	0.105	0.365	0.225	0.783	0.445
	rs17375748	0.270	0.399	0.193	0.542	**0.001**	0.659	0.729	0.140	0.616	0.208
	rs61822012	0.217	0.651	0.197	0.161	0.669	**0.004**	0.203	0.425	0.888	0.290
	rs2486253	0.908	0.427	0.318	0.453	0.575	0.076	0.404	0.773	0.222	0.396
	rs1186688	0.324	0.612	0.121	0.222	0.860	0.092	0.972	0.600	0.196	0.224
	rs12729701	0.453	0.282	0.499	0.309	0.233	0.126	0.637	0.769	0.489	0.822
	rs1890532	0.665	0.693	0.398	**0.015**	0.070	0.893	0.137	0.540	0.585	0.890
	rs1186689	0.324	0.984	**0.020**	0.536	0.417	0.084	0.072	1.000	0.712	0.436
	rs1186685	0.176	0.741	0.205	0.153	0.781	**0.006**	0.209	0.403	0.896	0.297
	rs12122979	0.251	0.225	0.437	0.512	0.591	0.056	0.372	**0.047**	0.902	0.629
KCNJ9	rs6677510	0.218	0.073	0.138	0.787	0.612	0.070	0.070	0.363	0.820	0.118
	rs2737702	0.863	0.641	0.603	0.506	0.324	0.862	0.909	0.449	0.870	0.363
	rs2737703	0.664	0.996	0.379	0.460	0.245	0.602	0.892	0.265	0.952	0.398
	rs2753268	0.509	0.238	0.213	0.368	0.231	0.429	0.437	0.134	**0.024**	0.172
	rs2494211	0.526	0.732	0.484	0.500	0.322	0.756	0.529	0.111	0.784	0.220

Linear regression using Pearson's r test. P value <0.05 is significant (shown in bold)
